# Effect of intraarticular pressure on glenohumeral kinematics during a simulated abduction motion: a cadaveric study

**DOI:** 10.1186/s12891-023-06127-0

**Published:** 2023-02-07

**Authors:** Patrick M. Williamson, Kaveh Momenzadeh, Philip Hanna, Mohammadreza Abbasian, Nadim Kheir, Aron Lechtig, Stephen Okajima, Mason Garcia, Arun J. Ramappa, Ara Nazarian, Joseph P. DeAngelis

**Affiliations:** 1 Musculoskeletal Translational Innovation Initiative, Carl J. Shapiro Department of Orthopaedic Surgery, Beth Israel Deaconess Medical Center and Harvard Medical School, 330 Brookline Avenue, Boston, MA RN115 USA; 2 Mechanical Engineering Department, Boston University, Boston, MA USA; 3 Carl J. Shapiro Department of Orthopaedic Surgery, Beth Israel Deaconess Medical Center, Harvard Medical School, Boston, MA USA; 4 Department of Orthopaedic Surgery, Yerevan State Medical University, Yerevan, Armenia

**Keywords:** Negative intraarticular pressure, Passive stabilizers, Glenohumeral joint, Glenohumeral joint capsule, Stability

## Abstract

**Background:**

The current understanding of glenohumeral joint stability is defined by active restrictions and passive stabilizers including naturally-occurring negative intraarticular pressure. Cadaveric specimens have been used to evaluate the role of intraarticular pressure on joint stability, although, while the shoulder’s negative intraarticular pressure is universally acknowledged, it has been inconsistently accounted for.

**Hypothesis:**

During continuous, passive humeral abduction, releasing the native intraarticular pressure increases joint translation, and restoring this pressure decreases joint translations.

**Study design:**

Descriptive Laboratory Study.

**Methods:**

A validated shoulder testing system was used to passively abduct the humerus in the scapular plane and measure joint translations for seven (*n* = 7) cadaveric specimens. The pressure within the glenohumeral joint was measured via a 25-gauge needle during passive abduction of the arm, which was released and subsequently restored. During motion, the rotator cuff muscles were loaded using stepper motors in a force feedback loop and electromagnetic sensors were used to continuously measure the position of the humerus and scapula. Joint translation was defined according to the instant center of rotation of the glenohumeral head according to the recommendations by the International Society of Biomechanics.

**Results:**

Area under the translation versus abduction angle curve suggests that releasing the pressure within the capsule results in significantly less posterior translation of the glenohumeral head as compared to intact (85–90˚, *p* < 0.05). Posterior and superior translations were reduced after 70˚ of abduction when the pressure within the joint was restored.

**Conclusion:**

With our testing system employing a smooth continuous passive motion, we were able to show that releasing intraarticular pressure does not have a major effect on the path of humeral head motion during glenohumeral abduction. However, both violating the capsule and restoring intraarticular pressure after releasing alter glenohumeral translations. Future studies should study the effect of simultaneous external rotation and abduction on the relationship between joint motion and IAP, especially in higher degrees of abduction.

**Clinical relevance:**

Thoroughly simulating the glenohumeral joint environment in the cadaveric setting may strengthen the conclusions that can be translated from this setting to the clinic.

**Supplementary Information:**

The online version contains supplementary material available at 10.1186/s12891-023-06127-0.

## What is known about this subject

The role of negative intraarticular pressure on joint function has been defined for static positions, but studies have not isolated its function during glenohumeral motion.

## What this study adds to existing knowledge

This study employs a novel system that performs a highly repeatable motion during different capsular conditions, including restoring the pressure within the joint post-release, to show the effect of negative intraarticular pressure on the motion path of the humeral head during abduction.

## Introduction

The glenohumeral (GH) joint has a wide range of motion, limited bony restriction, and is dependent on active and passive contribution for stability [1–4]. The GH joint capsule encompasses the joint and defines the volume of the joint space [5]. This soft tissue envelope establishes the negative intraarticular pressure (IAP), a passive contributor to maintaining stability [5–8].

A major component of GH research aims to understanding how negative IAP limits distraction and stabilizes the joint [8–11]. Preliminarily, radiographs were used to qualitatively assess joint distraction before and after venting the capsule in cadavers, [8] which has since been quantified with varying loads applied to the hand [11]. *In-vivo* studies established a broader understanding of the importance of negative IAP in healthy and injured shoulders, and showed that negative intraarticular pressure may not be present after some GH injuries [5, 9]. Overall, these studies were limited to joint distraction, which is crucial to the understanding of IAP, but do not describe the effect of IAP during the GH joint’s wide range of motion.

Although challenging, studies have also used cadavers to evaluate the role of IAP on GH joint translations during motion with and without simulated rotator cuff (RC) muscle activation [7, 12–16]. Notably, Hurschler et al. used a dynamic arm simulator to study IAP’s effect on translation by moving the humerus through elevation for different capsular conditions [7]. They found that venting both the subacromial bursae and the capsule to atmospheric pressure increased superior translation, but the effect of the GH capsule was not isolated, and IAP was not recorded [7]. Additionally, Inokuchi et al*.* measured IAP in cadavers during a number of motions [16]. With increasing abduction angle, they found an increase in IAP (toward atmosphere), but the muscles were not loaded, and translation of the humeral head was not reported [16]. These studies have begun to characterize IAP’s effect on GH motion, but GH IAP’s isolated effect on motion has not yet been quantified.

Though the GH joint’s negative IAP is universally acknowledged, cadaveric studies that evaluate function, injury, and repair inconsistently account for it. Many studies normalize the joint pressure in cadaveric specimens by releasing this pressure and simulating its function with external joint compression [13, 17–23]. Others maintain the IAP for the intact control condition and then release the joint pressure to simulate the injury or procedure [24, 25]. Controlling for the IAP in a cadaveric specimen is a known issue, but studies that intend to translate results from the cadaveric setting to the patient depend on a more robust definition of IAP’s role during GH motion.

The goal of this study was to evaluate IAP’s contribution to joint translations during a GH abduction motion performed in the cadaveric setting and establish a model that more accurately simulates GH motion. We also characterized the change in IAP during abduction for our testing system. We aimed to isolate the contribution of IAP to joint translations by releasing the IAP via needle decompression and subsequently restoring IAP to see if it’s contribution could be re-established. In this investigation, we employed a validated, cadaveric shoulder testing system, [26–28] and used joint motion definitions recommended by the International Society of Biomechanics [29] to study joint mechanics in a repeated measures manner. *We hypothesized that releasing the IAP in the joint would increase the joint translations in the superior and posterior directions. Also, we hypothesized that restoring IAP after releasing would restore GH joint translations (decrease superior and posterior translations).*

## Methods

### Sample preparation

Seven (*n* = 7) fresh-frozen intact left human shoulders (Medcure, Inc.) with mean age 76.9 years (range 56–90) were examined using a validated shoulder testing system with six degrees-of-freedom (DOF) [26, 27]. Four specimens were from female donors. Each shoulder specimen was inspected to ensure the integrity of the RC and joint capsule. In particular, the rotator interval was closely observed, while the joint was taken through a gentle range of motion examination. Only specimens that demonstrated an indentation in the rotator interval and a firm, well-centered shoulder were used. This gross assessment intended to confirm the joint’s integrity and the presence of normal negative IAP. The same researcher (PH) performed all specimen dissection and preparation. The absence of joint arthropathy was confirmed for all specimens after testing.

The skin and the superficial musculature were removed, while maintaining the integrity of the RC muscles and the joint capsule, and the clavicle was disarticulated at the acromioclavicular joint [14]. The remaining tissue was kept moist with physiologic 0.9% saline throughout testing [30, 31]. The RC muscles were elevated off of the scapula, and sutures were placed through the free ends between two layers of a nylon belt using a locking Krackow stitch. The shoulder was mounted by rigidly fixing the scapula with the center of the glenoid and the superior and inferior angles of the scapula aligned with the vertical plane. The scapula was then tilted 30˚ upward relative to the vertical [32] and 10˚ anteriorly to mimic the physiologic resting position of the scapula [7, 14, 33]. This position represents a reasonable static position for 90–120˚ of humerothoracic motion (60–90˚ of GH motion. This range of motion (60–90˚ of GH motion) is of specific interest, as exposure to overhead activities may increase the risk of shoulder injuries [34, 35].

### Testing conditions

Four IAP and capsular conditions were evaluated during continuous, passive abduction of the humerus. Specimens were tested with 20 N static RC load with 1) Intact Capsule, 2) Pressure-Released Capsule, 3) Pressure-Restored Capsule, and 4) Violated Capsule. IAP was released using the same 25-gauge needle used during IAP measurement. Similarly, IAP was restored with this needle and a 5 mL syringe connected to a lead of the pressure monitor.

### Testing system

**Fig. 1:**
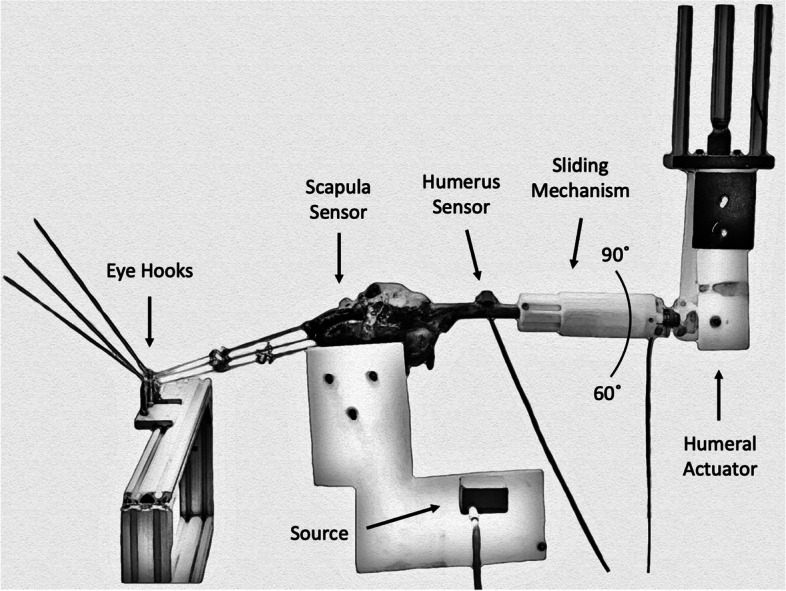
Cadaveric testing system. Eye hooks are used to direct the rotator cuff muscle lines of action and connect to stepper motors (not pictured) that actuate the rotator cuff muscle load. The electromagnetic position and orientation data collection system comprises the scapula sensor, humerus sensor, and source. The two-part sliding mechanism limits artificial compression of the joint without adding friction to the motion. The robotic arm applies continuous, repeatable motion to the humerus between 60˚ and 90˚ of scapular plane abduction

The previously validated testing system allows for actuation of the major RC muscles (Infraspinatus/Teres Minor unit, Subscapularis, and Supraspinatus) [26–28], Fig. 1. To ensure that GH motion was isolated, a closed-loop system with a proportional controller was created to actuate the RC muscles during motion. This system included a tensile load cell (LCM300, 50 lb, FUTEK Advanced Sensor Technology, Inc), 2 springs in parallel, and one bipolar stepper motor (Nema 23, Gear Ratio 47:1, STEPPERONLINE) per RC muscle. A custom MATLAB script was used to automatically adjust the load applied to each muscle during motion.

A two-piece humeral slide mechanism was implemented to minimize external joint compression applied by the humeral actuator during passive motion of the humerus. The humerus was potted using a two-part epoxy (Smooth-Cast 300q, PMC Smooth-on Inc) in one component. The second component was mounted on the actuator of the system. With linear bearings that significantly reduce friction between the components, this system allowed for smooth articulations between the humerus and the actuator.

### Abduction motion

Electromagnetic sensors were rigidly fixed to the scapular spine and the deltoid tuberosity of the humerus [11]. Using an electromagnetic motion tracking system (Liberty, Polhemus, VT), the 6 degree-of-freedom motion of the bone-embedded sensors was recorded at 120 Hz. Proper calibration was performed to ensure that metal components in the testing space did not interfere with data collection. Reference points on the humerus and the scapula, according to the recommendations by the International Society of Biomechanics, [29] were recorded at approximately 90˚ of abduction in the scapular plane. The instantaneous GH center of rotation was estimated using a calibration motion: passively rotating the humerus in the test space, then using a least squares algorithm to fit a sphere to that motion, as reported previously [12, 36]. Together, these points were used to create the humerus and the scapula coordinate systems, [29] and a custom MATLAB script was used to calculate and display the GH angles in real-time. This allowed for precise specimen positioning during testing.

With the feedback of the anatomical coordinate systems and the programmable humeral actuator, [37–39] the humerus was passively raised in the scapular plane from 60 to 90˚ of GH joint abduction, which represents approximately 90 to 120 humerothoracic abduction, for five repetitions after a single preconditioning trial. The humerus was fixed so the medial and lateral condyles aligned vertically at 90˚ humerothoracic abduction, and the system did not allow for external rotation of the humerus. To protect the specimens, testing was performed at a reduced speed (duration of abduction motion, 28 s).

### RC muscle activation

The RC muscles were then attached to the stepper motors by high tensile strength cord [40] and a series of eye hooks in the following manner: the supraspinatus line was adjusted to form a 10˚ angle below the horizontal line, the subscapularis line was adjusted at an angle bisecting the lateral margin and the spine of the scapula ventrally, and the infraspinatus line was adjusted in the same manner as the subscapularis, but dorsally [7]. This allowed for loading the muscles along their physiological lines of action as described previously [7] and shown in Fig. 1. The eyehooks were lubricated to minimize friction during loading.

The stepper motors were adjusted by proportional force control feedback to apply an equal static load (20 N) to each RC muscle throughout the arc of GH motion, i.e. the stepper motors adjusted the tension in line as the arm was abducted. A wide variety of RC muscle profiles have been used to study GH motion [7, 22, 41–43], so we chose a constant force profile that preserved the integrity of the capsule and supraspinatus. The constant force profile also allows for studying humeral abduction’s effect on IAP. The shoulder specimen was continuously inspected to ensure the integrity of the RC and joint capsule throughout the process of specimen preparation and testing.

### Joint translations

Translations of the instant center of rotation of the humeral head relative to the scapula coordinate system were reported as percentages relative to the dimensions of the glenoid. For translations in the anterior–posterior (AP) direction, translations are reported relative to the glenoid width (${W}_{glen}$) and superior-inferior (SI) translations are reported relative to the glenoid height (${H}_{glen}$). medial–lateral (ML) translations are reported relative to a combination of the height and width of the glenoid according to: $\frac{1}{2}({H}_{glen}+{W}_{glen})$

### Intraarticular pressure measurement

As an additional characterization of the relationship between GH abduction in the scapular plane and IAP, IAP was measured during discrete positioning of the humerus. After collecting kinematic data for the intact condition, the arm was fixed at 60˚ of scapular plane abduction, where a 25-gauge needle connected to a pressure monitor (PressureMat DPG, Pendotech) filled with saline was passed into the articular space through an entry point located in the infraspinatus tendon. This entry point was located 1 cm inferior and lateral to the posterior angle of the acromion process and directed towards the coracoid process anteriorly [11]. Other approaches to the joint space were attempted in a pilot study, but each approach compromised the capsule’s seal. The needle was removed during GH motion, so this approach likely did not affect the load applied via the infraspinatus. The native IAP was recorded, then communication with the joint space was confirmed by distracting the joint and confirming a reduction in IAP [23]. The effect of GH abduction on IAP was assessed by recording IAP while discretely positioning the humerus between 60˚ and 90˚ abduction at 5˚ increments. This procedure was repeated for three abduction motions and the IAP for each position was taking as the average of the three.

### Data analysis

GH translation in the superior, posterior, and lateral direction for each condition was recorded continuously throughout the abduction motion from 60 to 90˚ of scapular plane abduction. For each condition, the average of five repetitions of GH translation was plotted over time, and the area under the curve (AUC) for 5˚ increments was calculated to assess the path-dependent motion, as reported previously [31, 44–46].

Shapiro–Wilk test was used to assess the normality of the data for each variable. Data were analyzed using two-way repeated measure ANOVA, where "Abduction angle" and "Capsule condition" are within-subjects factors. The Fisher's LSD post-test followed analysis for multiple comparisons of simple effects of capsule condition on translation percentage within each abduction angle. *P* < 0.05 was considered as the significant level. All statistical analysis was performed using GraphPad Prism (version 8.4.3 for Windows; GraphPad Software, San Diego, CA).

## Results

### AUC for glenohumeral translations during scapular plane abduction

Analysis of the path-dependent motion was assessed by calculating the AUCs for 5˚ increments of abduction, Fig. 3 which showed increasing superior and posterior translations during abduction. After 70˚ of abduction, restoring the pressure within the joint decreased the translation in the posterior direction (*p* < 0.05) and violating the capsule increased the translations in both the posterior (*p* < 0.05) and superior directions (*p* < 0.05) relative to intact. The alteration to translation due to releasing the pressure within the joint was not significantly different from intact in any direction.

### IAP during scapular plane abduction

**Fig. 2:**
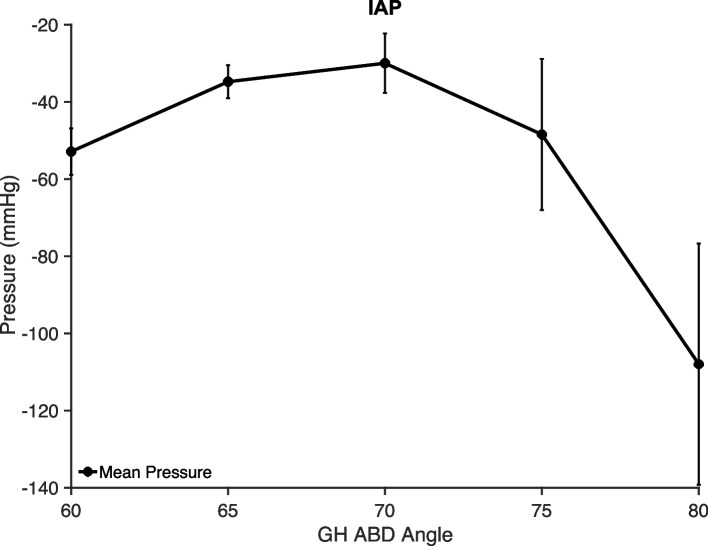
Average (± Standard Error) of three trials for each specimen of Intraarticular Pressure (IAP) between 60˚ and 80˚ of abduction in the scapular plane

The IAP within the GH joint was negative at 60˚ abduction in the scapular plane for all specimens with a mean of 46 (± 24) mmHg. With passive abduction of the arm, the IAP increased reaching its peak at 70˚ of abduction, as seen in Fig. 2. Further abduction led to a decrease in IAP that was most prominent from 75˚ to 80˚ of GH abduction.

## Discussion

The findings reported herein reveal that both abduction degree and capsule condition are closely related to the humeral head's translation in both the superior-inferior and anterior–posterior directions. Restoring the IAP pressure was found to decrease the GH joint translations in the posterior direction between 75˚ and 90˚ of abduction (*P* < 0.05), supporting our hypothesis, though there was no difference in superior translation between the intact capsule and the pressure restored conditions. When releasing the IAP from the GH joint, no increase in the joint translation was seen in the superior or posterior directions. Contrary to the expected outcome, a statistically significant decrease in superior translation was seen in the final range of abduction (85˚-90˚). These differences were not statistically significant in other directions (superior-inferior, lateral, or total translation). The alteration to joint translation between capsule conditions is less than expected and may result from the limited range of motion applied in this study.

### Glenohumeral translation changes with IAP during scapular plane abduction

**Fig. 3:**
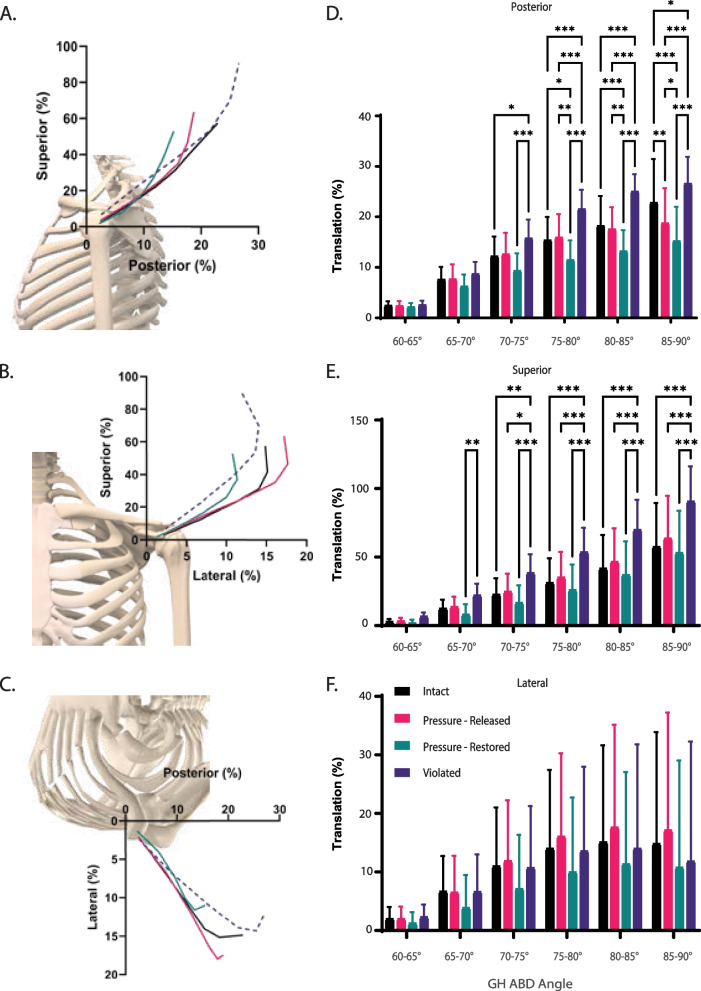
Humeral head translation for three capsular conditions: intact, pressure released, and pressure restored during scapular plane abduction between 60˚ and 90˚. Anterior, superior, and lateral translations were taken as positive. Anatomical pictures are not to scale. Figures **A**-**C** show the average 2D projection of the motion of the humeral head in the **A** sagittal plane, **B** coronal plane, and **C** transverse plane. Figures **D**-**F** show the median (75th and 25th quartile) area under the translation versus abduction angle curve (AUC) for 5˚ increments of motion. * *p* ≤ .05, ** *p* ≤ .01, and *** *p* ≤ .001

In general, the instantaneous center of rotation translated posteriorly, superiorly, and laterally as the humerus was abducted in the scapular plane between 60˚ and 90˚, Fig. 3. We found modest changes in GH translation after altering the pressure within the joint. The AUC analysis revealed that posterior translation was reduced at the end of motion (85–90˚) for the pressure-released capsule in comparison to the intact capsule. However, the total magnitude of translation was not altered. This suggests that by releasing the IAP, the motion path may be changed. It is important to note that the motion simulated in this study is simple, slow, and highly repeatable. For more complex or dynamic movements, the IAP may play a more significant role should the active stabilization provided by the RC muscles not be sufficient to limit translation [47, 48].

Previous cadaveric work aiming to evaluate the effect of IAP on glenohumeral motion did not include conditions that isolated the effect of IAP. Hurschler et al*.* demonstrated that simultaneous release of IAP and pressure within the subacromial bursae increased translation of the GH head during elevation by 2.8 mm (S-I) and 1.1 mm (A-P) at 90˚ of scapular plane abduction compared to intact. Taking their convention, namely superior and anterior translations positive, we found a similar change 2.9 mm in the S-I plane, but opposite results of -2.31 mm in the A-P plane at 90˚. However, it should be noted that Hurschler et al*.* vented the subacromial bursae before the joint capsule and therefore could not comment on the specific effect of the capsular IAP on the translation of the joint [7].

Considering the results of the study by Hurschler et al*.* and studies that have quantified the distraction of the joint, we expected a significant effect of IAP on joint translation. The change, however, was only marginal. Hurschler et al*.* loaded the deltoid and linearly increased the forces applied to the RC muscles until they reached an estimated physiological RC force [7], while our system provides passive GH abduction via a humeral actuator, and the load we applied on the RC muscles (20 N each) was constant, similar to previous glenohumeral cadaveric studies [22, 41, 49]. A significant advantage of our system is that it employs a smooth, continuous passive humeral motion. After calibrating the specimen, a motion file specific to each specimen's geometry and exact position in the system can be created. This allows for multiple trials of precisely the same motion and the ability to simultaneously and continuously collect position data. An exciting and necessary next step would be to release only the pressure within the GH joint and perform the same abduction motion with active muscle activation provided by the deltoid and RC muscles with simulated arm weight.

There was a more considerable effect observed with re-establishing the pressure within the joint. By attempting to re-establish the pressure within the joint, we were essentially testing the reversibility of this process. Our results showed a statistically significant decrease in translation in posterior direction and total translation in 75–90˚ of abduction, compared to both intact and pressure-released conditions. These findings were not significant in the SI direction. Consequently, we found that we could not recreate the intact kinematics by re-establishing the native IAP in the joint which may be explained by the increased friction due to the loss of synovial fluid as the lubricant and capsular tightness result from losing moisture and flexibility.

Another important finding in our study was the increased humeral head translation in all directions after violating the inferior capsule, which was significant after 70˚ of abduction. This seems to confirm the hypothesis that the inferior capsular redundancy disappears after 70˚, and that the inferior capsular tightness, which is remarkable at this degree of abduction, reduces humeral head translation in superior, lateral, and spatial directions [50, 51]. Therefore, we hypothesize that there is a relationship between IAP, capsular tightness, GH abduction, GH external rotation, and RC load not fully captured by this study that defines the role of each on a joint translation during motion. Further studies are needed to evaluate this hypothesis.

### IAP during scapular plane abduction

Though the initial increase of IAP toward atmospheric pressure with increased abduction angle seen in this study has been has been shown previously [16, 52], the negative trend after about 70˚ has not been described. The reduced pressure between 70 and 80˚ measured in this study may be due to the status of the capsular tissue and the proximity of the greater tuberosity to the acromion. This is congruent with Pagnani et al*.*'s assertion that, at extremes of motion, capsular structures are wound up and pull the joint surfaces together [53]. Further, in a patient, the humerus externally rotates with increased abduction due to tension in the capsule [54–56]. Our system maintained vertical alignment of the medial and lateral epicondyles during the abduction motion. It, therefore, did not externally rotate the humerus between 80 and 90˚, which may have encouraged tightened capsular tissue and a decrease in IAP. Further studies are needed to define the relationship between abduction, external rotation, and negative IAP, especially as the arm approaches 90˚ of GH abduction.

Our native IAP measurements are comparable to those reported in some studies [5, 11, 16, 23]. It is important to note that our measurements were performed at 60˚ of scapular plane abduction, while some studies measured a single value with the arm at the side. Inokuchi et al*.* reported that the IAP approached atmospheric pressure between 0˚ and 60˚ of abduction [16], and further, we found that a disturbance to the arm's position could alter the pressure reading.

### Limitations

The methodological limitations of this study include those shared by all cadaveric investigations [2]. These include using dead tissue to simulate human motion, a static scapular position, and single lines of action for muscle activation. The application of the abduction motion, the simulated muscle activation, and IAP measurement have limitations specific to this study. The abduction motion applied by the humeral actuator was calibrated to each specimen, and a custom sliding cup was developed to minimize extraneous compression of the joint. Simulated RC muscle activation was considered constant during motion, which is unlikely to be the case in-vivo, and the muscle moment arms were not considered. This allowed for a direct comparison between conditions and evaluating how the degree of abduction altered the IAP. Also, this cadaveric model is limited to the RC and therefore does not include the deltoid or biceps, which may also contribute to GH joint translation. Saline entering the joint during IAP release may have affected the joint's kinematics [16], but because the range of motion tested was small and was performed slowly, this effect is likely insignificant. The lack of external rotation during abduction limits our approach, but most cadaveric studies of GH abduction share it. Since it was not possible to examine for labral lesions or PASTA without opening the capsule, we did not do so, however we recognize with age this could be an important structural change. Subsequent studies should study the effect of simultaneous external rotation and abduction on the relationship between joint motion and IAP, especially in higher degrees of abduction and evaluate the effects of the long head of the biceps on GH joint kinematics.

## Conclusion

With our testing system that employs a smooth, continuous passive motion, we were able to show that releasing intraarticular pressure does not have a major effect on glenohumeral translation during glenohumeral abduction. However, both violating the capsule and restoring intraarticular pressure after releasing alter glenohumeral translations. Future studies should study the effect of simultaneous external rotation and abduction on the relationship between joint motion and IAP, especially in higher degrees of abduction.

## Supplementary Information

**Additional file 1.**

## Data Availability

All data generated during this study are included in this published article or as supplementary information files.
